# Forced monogamy in a multiply mating species does not impede colonisation success

**DOI:** 10.1186/1472-6785-14-18

**Published:** 2014-06-12

**Authors:** Amy E Deacon, Miguel Barbosa, Anne E Magurran

**Affiliations:** 1 Centre for Biological Diversity, School of Biology, University of St Andrews, St Andrews, Fife KY16 9TH, UK; 2 CESAM, Department of Biology, Universidade de Aveiro, Campus de Santiago, 3810 Aveiro, Portugal

**Keywords:** *Poecilia reticulata*, Polyandry, Invasive species, Mesocosms, Population viability

## Abstract

**Background:**

The guppy (*Poecilia reticulata*) is a successful invasive species. It is also a species that mates multiply; previous studies have demonstrated that this strategy carries fitness benefits. Guppies are routinely introduced to tanks and troughs in regions outside their native range for mosquito-control purposes, and often spread beyond these initial confines into natural water bodies with negative ecological consequences. Here, using a mesocosm set up that resembles the containers into which single guppies are typically introduced for mosquito control, we ask whether singly-mated females are at a disadvantage, relative to multiply-mated females, when it comes to founding a population. Treatments were monitored for one year.

**Results:**

A key finding was that mating history did not predict establishment success, which was 88% in both treatments. Furthermore, analysis of behavioural traits revealed that the descendants of singly-mated females retained antipredator behaviours, and that adult males showed no decrease in courtship vigour. Also, we detected no differences in behavioural variability between treatments.

**Conclusions:**

These results suggest that even when denied the option of multiple mating, singly-mated female guppies can produce viable populations, at least at the founder stage. This may prove to be a critical advantage in typical introduction scenarios where few individuals are released into enclosed water bodies before finding their way into natural ecosystems.

## Background

A key criterion for invasion success is the ability of an invading population to grow to the point at which it becomes viable. To achieve this, it is essential that individuals do not lose behavioural traits important to survival, or behavioural variation in such traits [1-4]. Recent evidence suggests that individuals with greater variability in behavioural traits are more likely to establish viable populations in an invasion scenario [5]. Furthermore, founder populations of invading species are often small [6], limiting intra-specific encounters and consequently the benefits of mating with multiple partners. Here we ask whether the colonization ability of a multiply mating invasive species is impaired by forced monogamy, as may occur in an introduction situation.

The guppy (*Poecilia reticulata*) is established in at least 69 countries outside its native range of Trinidad and north-eastern South America [7]. Mosquito control programmes and the release of unwanted pets contribute equally to its non-native distribution [7]. Both routes typically involve initial introductions of a single fish or a few individuals to confined waterbodies such as tanks and troughs [8], although occasionally guppies may be released directly into natural streams and rivers.

The reproductive biology of this live-bearing species, particularly its ability to store sperm, helps explain its success in invading new habitats. A female guppy can produce repeated broods over a period of several months without the need for re-mating [9]. Indeed, single females collected from the wild and housed for one year in small water tanks – similar to those into which guppies are typically placed for purposes of mosquito control - establish viable populations in around 90% of cases [7].

A second variable, that may be important during invasion but has not been examined previously in this context in guppies, is multiple mating. Female multiple mating is ubiquitous in guppies [10,11]. In fact, guppies hold the highest total number of putative sires per brood recorded for a vertebrate species [12]. Multiply-mated females have been shown to produce larger broods [13,14], with greater levels of genetic and phenotypic variability [15,16]. All of these multiple mating benefits could be advantageous in an introduction scenario, where founding numbers are often low [17]. For example, by giving birth to multiply-sired broods, potential demographic bottlenecks may be minimised, and more variation maintained in the resulting population [18].

A typical invasion sequence begins with the transportation and introduction of a small number of individuals from their native range to a new environment [6]. The vast majority of introduction events will fail to result in an invasion [19,20]. A key criterion for invasion success is the ability of an invading population to grow to the point at which it becomes viable. A viable population being one that establishes successfully, and in which the behavioural performance and versatility of the progeny is not degraded [5]. As invasion events tend to involve only a few individuals, females may not have the opportunity to mate with more than one male. It is therefore important to examine whether females who have not had access to multiple males are less successful in founding a viable population during invasion.

Two aspects of behavioural performance relevant here are the survival abilities of offspring, and the mating vigour of males. Offspring are born with innate abilities to school with conspecifics, cautiously inspect novel stimuli and display an escape response when threatened [14,21]. Male guppies engage in a range of courtship behaviours, including consensual sigmoid displays and coercive ‘sneaky’ gonopodial thrusts [22]. Previous studies have found evidence of reduced courtship vigour and mating success in male guppies after 1-3 generations of inbreeding [23,24]. To produce a viable population it is essential that the individuals produced in the new habitat maintain the ability to respond to the threat of predation, recognise potential mates and adjust their mating behaviour according to the context [25]. Failure to retain these behaviours, and variability in these behaviours, can lead to reproductive failure [26] and may decrease the chances of success of a newly established introduced population [5].

In this study we ask whether guppy females can successfully found a population at the outset of an invasion, when mating partners are scarce and forced monogamy is their only option. We report the results of an experiment in which we measure the ability of both singly-mated and multiply-mated females to found viable populations. We also examine the magnitude and variability of the behavioural responses of the progeny produced under these two treatments. This experiment is designed to mimic the conditions that prevail in the earliest stages of an invasion when single or very small groups of guppies are released into containers or small pools where no other fish are present.

## Results

### Population establishment

**Figure 1:**
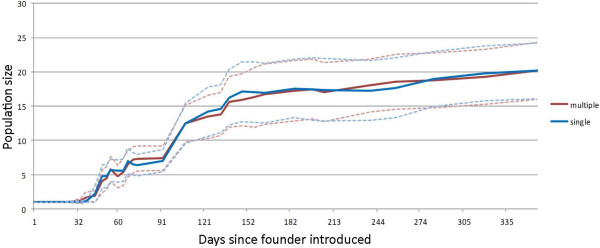
**Mean growth trajectories for single and multiple mated mesocosms over 12 months showing cumulative numbers of individuals.** Dotted lines denote 95% confidence intervals.

Of the 40 mesocosm tanks, 35 (87.5%) successfully established populations that still persisted one year after initial introduction. Of the five extinctions, three were from the single and two from the multiple mating treatments. In all cases, the extinction was due to a failure of the female to establish a population at all; no offspring were ever recorded in these five tanks. Population growth trajectories from the visual census data show no difference between the treatments (Figure 1). One year after establishment, the full census also showed no significant difference in final population size between those founded by singly or multiply-mated females (t = 0.504; df = 33; p = 0.618).

### Male colouration diversity

Mesocosm populations from the ‘multiple’ mating treatment showed significantly greater between-fish complementarity in colour patterns (F_1,30_ = 6.432; p = 0.017), thus suggesting that multiple mating had indeed led to multiple paternity in this treatment.

### Behavioural performance and variation

**Figure 2:**
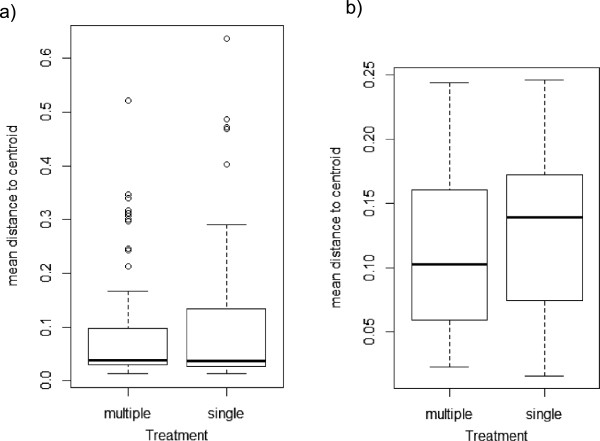
**Beta-dispersion analyses. (a)** Juvenile behavioural performance **(b)** male mating behaviour.

There was no significant difference in the antipredator performance of newborns descending from single or multiple mesocosms (MANOVA: F_8,28_ = 1.517; p = 0.196; Table 1), or in the mating behaviour of male descendants (MANOVA: F_3,30_ = 1.884; p = 0.154; Table 2).The results from the multivariate analysis of dispersion for juvenile antipredator performance revealed no significant variation within or between mating treatments (Permutation test: p = 0.253, p = 0.077, respectively). In terms of male mating behaviours, the analyses revealed a significant difference in variation in male mating behaviours between mesocosms (Permutation test: p = 0.003) but not between mating treatments (p = 0.286) (Figure 2).

**Table 1:** MANOVA analysis of juvenile behavioural performance, with treatment (singly or multiply-mated female founded populations)

**Multivariate tests**	**Wilks’ λ**	**Df**		**F**	**p**
***Treatment (single or multiple)***	0.698	8, 28		1.517	0.196
**Between-subjects tests**	**SS**	**Df**	**MS**	**F**	**p**
***Treatment (single or multiple)***					
Schooling	67.707	1	67.707	0.035	0.853
Evasion	2.753	1	2.753	1.445	0.237
Time in cover	9.884	1	9.884	0.005	0.947
Activity	1106.139	1	1106.139	7.598	0.009**
Reaction distance	2.865	1	2.865	0.728	0.399
Mean inspection distance	0.088	1	0.088	0.018	0.895
Inspection frequency	33.141	1	33.141	1.082	0.305
% inspections alone	235.361	1	235.361	0.836	0.367
***Error terms***					
Schooling	68223.616	35	1949.246		
Evasion	66.674	35	1.905		
Time in cover	75868.571	35	2167.673		
Activity	5095.301	35	145.580		
Reaction distance	137.706	35	3.934		
Mean inspection distance	174.754	35	4.993		
Inspection frequency	1071.942	35	20.627		
% inspections alone	9850.451	35	281.441		
***Total***					
Schooling	2033826.300	37			
Evasion	811.253	37			
Time in cover	1287814.099	37			
Activity	29106.983	37			
Reaction distance	1182.704	37			
Mean inspection distance	2137.528	37			
Inspection frequency	14585.868	37			
% inspections alone	2137.528	37			

**significant at the 1% level.

**Table 2:** MANOVA analysis of male mating behaviour, with treatment (singly or multiply-mated female founded populations)

**Multivariate tests**	**Wilks’ λ**	**Df**		**F**	**p**
***Treatment (single or multiple)***	0.841	3, 30		1.884	0.154
**Between-subjects tests**	**SS**	**Df**	**MS**	**F**	**p**
***Treatment (single or multiple)***					
Following	2636.374	1	2636.374	0.200	0.658
Sigmoids	6.989	1	6.989	0.686	0.414
Thrusts	1.682	1	1.682	0.111	0.741
***Error terms***					
Following	422216.710	32	13194.272		
Sigmoids	326.173	32	10.193		
Thrusts	484.699	32	15.147		
***Total***					
Following	1128691.983	34			
Sigmoids	612.232	34			
Thrusts	1090.770	34			

## Discussion

Exotic species often find themselves in very small numbers in the early stages of invasion, which can limit mating opportunities [27,28]. For a species such as the guppy, which ordinarily employs a strategy of multiple mating, it might be expected that such forced monogamy would hinder success. However, in this study we found no evidence that single-mating impeded the colonisation success of female founders. This suggests that multiple mating is not critical to the invasion success of the guppy. Instead, it may be the degree of variability in mating strategies that contribute to their remarkable global success.

Several studies have shown that multiply-mated females produce larger broods with greater viability than singly-mated females [29,30]. These benefits could influence the success of establishing new viable populations. Our results, however, reveal that forced monogamy did not affect the chance of establishment or behavioural viability in our guppies. Both treatments showed extremely high establishment success rates after 12 months (88%) and there was no difference in growth trajectory or population size. Guppies are plastic in response to environmental changes, including life history traits such as number of offspring [31]. A difference between our study and earlier work is that previous studies have been designed to track each generation and compare within a generation [13,14]. The design of our study meant that the offspring we were comparing could potentially range between F1 to F4. The purpose here was to compare freely breeding populations, to mimic an introduction scenario, but this may also have added variability to both treatments. For this reason, we should be cautious in making direct comparisons between our results and those of previous studies.

The remarkable establishment success of the guppy, and specifically the ability of single females to found viable populations, has been previously demonstrated with wild caught fish [7]. Here, we have taken this a step further by manipulating the mating opportunities of the founding females, replicating an early-stage introduction scenario in which the Allee effect is predicted to act against success [32,33]. Allee effects describe any reduction in individual fitness as a consequence of low population density [34]. The lack of mating opportunities when population size is low is one such effect [32], and for introduced guppies may mean that the usual strategy of multiple mating is no longer an option. As well as benefits of multiple mating, potential costs also exist [14]. For example, the female in our multiple treatment may have experienced higher levels of harassment and/or more mating attempts than the female who was presented with the same male each day [35]. However, it is generally accepted that the benefits of multiple mating to the female tend to outweigh such costs. Our results indicate that even those females denied the option of multiple mating, and consequently the associated fecundity benefits, can still found viable populations. This suggests that female multiple mating is not critical to colonization success and does not reduce behavioural viability, at least in the initial establishment stages of the invasion process.

Furthermore, behaviours critical to survival and viability were maintained in these populations; twelve months after the female founder was introduced, offspring displayed antipredator behaviours at a similar frequency in both treatments. Similarly, male descendants (1-3 generations from the founder female) from both treatments courted females with equal levels of vigour.

Despite the genetic basis for courtship traits appearing to leave little scope for the manifestation of severe inbreeding effects [24], previous studies have demonstrated that between 1 and 3 generations of full-sibling inbreeding can be sufficient to detect reductions in male courtship vigour in the guppy [23,24] and other poeciliid species [36], specifically in terms of frequency of sigmoid displays and time spent following females. We, however, found no evidence of this in our mesocosm populations. Because we did not track generation in our experiment, it is possible that the levels of inbreeding (somewhere between 1 and 3 generations) were insufficient to cause behavioural differences. Ours is not the only study that did not find evidence of inbreeding effects on male mating behaviour in guppies [37], and it has been shown that rather than relatedness, the type and frequency of mating behaviour employed by male guppies is highly plastic in response to social and environmental cues [38]. Tested males came from populations with similar environmental and social conditions, which is likely to play a more important role than the treatments in determining variation in sexual behaviour [39]. It is also possible that genetic bottlenecks in the history of the laboratory population may have served to purge deleterious alleles, resulting in a natural tolerance for high levels of inbreeding [37,40].

By retaining antipredator behaviours and courtship behaviours despite descending from just two individual founders, these populations are well equipped for survival, persistence and potential invasive spread. This is of particular relevance to a typical introduction scenario where few individuals are introduced into a well or trough for mosquito control purposes. The speed at which the mesocosm populations established and grew suggests that such populations are likely to be well established by the point at which they find an opportunity to escape to natural water bodies, for example during floods or monsoon rains [8], and our findings indicate that at this stage they will have retained the behaviours that facilitate their survival.

In addition to the magnitude of behavioural performance, extent of behavioural variation was also the same in both treatments. Maintaining greater within-brood variation has been postulated as one of the potential advantages of a multiple mating strategy in an introduction situation, as it could maximise the variation on which natural selection could act – especially in a novel or changing environment [5,17]. However, we found no evidence of reduced behavioural variation in the singly-mated treatment, despite the fact that we did detect reduced colour-pattern variation in the males from these tanks. That variation is maintained in behavioural traits despite the loss of genetic variation at this early stage of invasion is impressive, and may be relevant to the success of the guppy as an invasive species. It has recently been demonstrated that phenotypic diversity in guppies is maintained through negative frequency-dependent selection in which rare male phenotypes are favoured [16,41]. This mechanism could help explain the lack of differences in the behavioural dispersion between males from the two treatments.

Our study used guppies descended from a population historically exposed to ‘high predation’ conditions. Although previous work indicates that evolutionary history linked to predation regime is not critical to establishment success [7], guppy populations are diverse and all will not necessarily show the responses documented here.

## Conclusions

Multiple mating does not appear to be critical to colonisation success in the guppy. Even when founders are restricted to just one male and one female, resulting populations thrive, at the colonization stage at least. Instead, it seems likely that rapid initial population growth is critical as it may both help minimize loss of behavioural variation as well as increasing propagule pressure early on in the invasion process [42-44]. These findings hold particular interest for scenarios, such as those commonplace in Southern India and parts of Africa [8], where guppies and other poeciliids are being introduced into water containers for mosquito control. Here, it is the initial stage of population establishment and persistence which is critical to their ability to spread into natural ecosystems at a later date – sometimes with disastrous consequences.

## Methods

We used fish descended from the Lower Tacarigua River in Trinidad. Virgin females (N = 40, mean total length 26.4 mm ± SD1.03) were placed in individual mesocosms (450 × 300 × 250 mm), set up with gravel, plastic plants, large pebbles and Java moss, and filled with dechlorinated water. Females were left to settle for 72 hours. After this acclimation period, a single male (N = 100, mean total length 21 mm ± SD1.42) was haphazardly introduced to each of the 40 mesocosms. All males were removed after 24 hours. In half of the tanks (N = 20) the same male was again introduced to the same mesocosm he had been in the day before (treatment 1: ‘single’), while in the remaining mesocosms (N = 20), a different male was introduced (treatment 2: ‘multiple’). This procedure was repeated for four days, with the end result that in the ‘single’ treatment the female had access to the same male for four consecutive days, whereas in the ‘multiple’ treatment the females had the opportunity to mate with four different males. At the end of the fourth day, all males were removed. Females and their resulting populations were fed daily on a diet of flake food, and were allowed to reproduce and establish for 12 months. It is a reasonable assumption, given the full 24 hour period and what we know about guppy sexual behaviour [11], that females will have had some kind of sexual contact with all four males.

Fish were allowed to mate freely within the mesocosms, which means that descendants used in the behavioural trials cannot be designated to a particular generation. However, guppy life-history is well known [9], and based on a three month period between birth and sexual maturity, we can estimate that juveniles tested were between 1 and 4 generations from the founder fish, and that adult males were between 1 and 3 generations from the founders.

### Population establishment

All mesocosms were inspected daily for new born individuals. After 12 months, a full census was conducted by collecting and counting every fish from each mesocosm.

### Male colouration diversity

During the 12 month census, each sexually mature male from every mesocosm was carefully placed in a zip-lock bag filled with a small amount of water, and photographed on its side.

### Behavioural performance and variation

#### Newborn antipredator behaviour

After the 12 month census, sexually mature females from all established mesocosm populations were isolated in individual tanks and allowed to produce a brood. Tanks were labelled according to an arbitrary code with corresponding key to enable ‘blind’ testing. Within 48 hours of birth, each newborn was tested in a series of behavioural assays. Schooling (N = 45), evasion ability (N = 54), time in cover (N = 54), activity (N = 54), reaction distance (N = 54) and inspection behaviour (N = 39). Each fish experienced the assays in this order as it made it easier to track individual fish. Where pairs of fish were tested (schooling and inspection behaviour), siblings were always used.

Schooling, evasion ability, time in cover, activity and reaction distance were tested using previously published assay protocols [7]. Following these tests, two fish were gently transferred two at a time to the inspection arena. This was the same tray used earlier for schooling, but filled to a deeper level with water (3 cm) and lined with graph paper underneath the base. A coloured, plastic object was positioned at the back edge of the tray. The two fish were released at the opposite end of the arena and allowed to roam for 10 minutes. During this time, the number of inspections (‘frequency’), proximity of approach during each inspection (‘mean distance’) and group size at approach (to calculate ‘% alone’) were all recorded. Inspection behaviour was conspicuous, and consisted of a directional approach towards the object, followed by a sideways glide and then retreat [45].

After this, all offspring were returned to their original mesocosms.

#### Male mating behaviour

After the 12 month census, the frequency of sexual behaviour of sexually mature males from the mesocosms was recorded. Each focal male was introduced to an observational tank containing three males and four females (these individuals were collected from a stock tank). During a 20-minute observation period the number of sigmoid displays and gonopodial thrusts were recorded, as well as the time (in seconds) the male spent following females. At the end of the observation period the focal male was returned to his original mesocosm.

No permits were required for these experiments. All behavioural observations were carried out at the School of Biology at the University of St Andrews. The premises where the observations were carried out comply with the ASAB Guidelines for the Treatment of Animals in Behavioural Research and Teaching, set by the UK Home Office (PCD 60/2609).

### Statistical analysis

#### Population establishment

To test whether mating treatment had an effect on the probability of establishing a viable population we compared the number of individuals produced between singly and multiply-mated founding populations using a *t*-test.

#### Male colouration diversity

To measure colour diversity within tanks we adapted a method from ecology used to assess the degree of variability, termed ‘complementarity’, in the species present in a group of sites [46]. Higher values of the index (C, for complementarity) mean that sites are more variable in terms of the species they support. Here, instead of using species presence/absence data, we considered the presence/absence of orange and black colour markings. As the complementarity analysis considers presence/absence as opposed to area or brightness, we restricted our analysis to the pigment colours (orange and black). These are present as easily identifiable, discrete markings, rather than in patches spanning large areas of the body, which is often the case for the iridescent structural colour markings (blue and yellow) [47,48].

**Figure 3:**
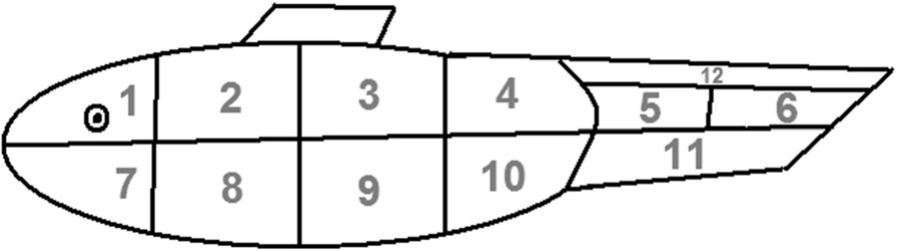
**Male body plan.** For each male, the presence/absence of orange and black colour markings was recorded for each of the twelve numbered sections.

A schematic body plan of a male guppy was split into 12 sections (Figure 3). The regions on the tail were designated in order to capture maximum variation in common markings. There was no *a priori* rationale behind selecting the regions on the main body; the body plan was simply divided up as evenly as possible.

The presence/absence of both orange and black colour markings (‘spot types’) within each section for each male fish was recorded from the photographs– creating 24 possible spot types. Within a mesocosm, each male was compared with every other male and the number of spot types unique to either fish was divided by the total number of spot types present for both fish combined:

(1) $$C_{\mathrm{jk}}=U_{\mathrm{jk}}/S_{\mathrm{jk}}$$

Where S_*jk*_ denotes the total number of spot-types possessed by fish j and k combined and U_*jk*_ is the number of spot-types unique to either fish *j* or *k*. The mean ‘*C*_*jk*_’ for all possible comparisons within each mesocosm was calculated and presented as the complementarity score (*C*). Higher values of ‘C’ reflect greater between-fish distinctness within each mesocosm population.

We used an analysis of co-variance to test for differences in within-mesocosm complementarity between treatments, using the number of males as a covariate. The hypothesis tested was that multiply-mated mesocosms would possess greater between-fish differences, reflected in higher complementarity scores (N = 16 for each treatment). Mesocosms containing <3 males were omitted from analyses.

#### Behavioural performance and variation

We used a multivariate analysis of variance (MANOVA) to examine behavioural performance in newborn offspring from the two treatments. A first MANOVA examined differences in schooling, evasion ability, time in cover, activity, reaction distance, % alone, frequency and mean distance between offspring from the two treatments. A second MANOVA examined differences in three mating behaviours (time spent following, sigmoid displays and gonopodial thrusts).

To explore the extent of adult and juvenile behavioural variation within mesocosms and whether this variation is affected by treatment, we estimated the phenotypic diversity in behavioural traits using a multivariate analysis approach. Number of chases, sigmoid displays and gonopodial thrust were used as adult behavioural variables. For juveniles we used schooling, evasion ability, reaction distance, activity time and time in cover as variables (see Additional file 1). These two groups of variables were used to calculate phenotypic similarities among individuals for males and juveniles respectively. We used these variables to build a similarity matrix by using Gower distance, as this distance measure is recommended for variables different in nature, as is the case here [49]. To avoid any variable dominating the distance measured, variables were standardized by dividing by the range, before computing the similarity matrix, thus ensuring that all variables had the same scale [50,51]. Individuals were then mapped into Euclidean multivariate space by implementing a Principal Coordinate Analysis (PCoA). This allowed us to calculate the position of the centroid (the spatial mean) of each group, and the distance of each individual to its group centroid. Phenotypic diversity was estimated for each colony and treatment as the mean distance to the group centroid in multivariate space [50]. We compared the distances of each individual to its group centroid to test for differences in phenotypic diversity among groups using a permutation test. The permutation test was run because of the inherent problems of ANOVA with the violation of multivariate normality [52]. The permutation test uses the same null hypothesis as the ANOVA, in that differences in phenotypic dispersion between the two groups of individuals are no more different than would be expected due to random chance at a level of probability of 5%. In the permutation test the least-squares residuals of the dispersion matrix were randomly re-shuffled 999 times. This generated a frequency distribution for the F-statistic under the null hypothesis of no difference in dispersion between phenotypes. Results were considered significant if the observed F statistic was greater than 95% for this frequency distribution (for *α* = 0.05).

All analyses were performed using SPSS v.19.0 [53], with the exception of the multivariate dispersion analysis which was performed using R [54].

## Competing interests

The authors declare that they have no competing interests.

## Authors’ contributions

AED and AEM were responsible for the conception and design of the study. AED carried out the experiments. AED and MB performed the statistical analysis. All authors helped draft the manuscript. All authors read and approved the final manuscript.

## Supplementary Material

Additional file 1 Table of means and SDs for newborn antipredator behaviours and male mating behaviours. Click here for file
