# Supplementary material for: Forced monogamy in a multiply mating species does not impede colonisation success
Source: BMC Ecol. 2014 Jun 12;14:18. doi: 10.1186/1472-6785-14-18 (PMC4067062; doi:10.1186/1472-6785-14-18)
Supplement: Additional file 1 — Table of means and SDs for newborn antipredator behaviours and male mating behaviours. [file 1472-6785-14-18-S1.doc]

**Additional File 1.**

Raw means and standard deviations (SD) for each behavioural variable.

| **Response variable** | **Treatment** | **Mean** | **SD** |
| --- | --- | --- | --- |
| *Newborn antipredator behaviours* | | | |
| Evasion (seconds) | Single | 4.85 | 2.82 |
|  | Multiple | 4.85 | 2.47 |
| Reaction (cm) | Single | 5.98 | 2.99 |
|  | Multiple | 5.11 | 2.46 |
| Activity (lines crossed) | Single | 28.91 | 15.70 |
|  | Multiple | 19.51 | 13.09 |
| Cover (seconds) | Single | 176.76 | 64.01 |
|  | Multiple | 177.52 | 76.35 |
| Schooling (seconds) | Single | 228.87 | 57.72 |
|  | Multiple | 226.19 | 59.84 |
| *Male mating behaviours* | | | |
| Sigmoids | Single | 3.13 | 6.01 |
|  | Multiple | 2.16 | 4.07 |
| Thrusts | Single | 1.41 | 2.41 |
|  | Multiple | 1.23 | 2.09 |
| Chasing (seconds) | Single | 140.40 | 156.52 |
|  | Multiple | 149.77 | 177.93 |
